# Non-Selective Cannabinoid Receptor Antagonists, Hinokiresinols Reduce Infiltration of Microglia/Macrophages into Ischemic Brain Lesions in Rat via Modulating 2-Arachidonolyglycerol-Induced Migration and Mitochondrial Activity

**DOI:** 10.1371/journal.pone.0141600

**Published:** 2015-10-30

**Authors:** Angela M. A. Anthony Jalin, Maheswari Rajasekaran, Paul L. Prather, Jin Sun Kwon, Veeraswamy Gajulapati, Yongseok Choi, Chunsook Kim, Kisoo Pahk, Chung Ju, Won-Ki Kim

**Affiliations:** 1 Department of Neuroscience, College of Medicine, Korea University, Seoul, Republic of Korea; 2 Department of Pharmacology and Toxicology, University of Arkansas for Medical Sciences, Little Rock, AR, United States of America; 3 Department of Biotechnology, School of Life Sciences and Biotechnology, Korea University, Seoul, Republic of Korea; 4 Department of Nursing, Kyungdong University, Wonju, Kangwon-do, Republic of Korea; Indian Institute of Integrative Medicine, INDIA

## Abstract

Growing evidence suggests that therapeutic strategies to modulate the post-ischemic inflammatory responses are promising approaches to improve stroke outcome. Although the endocannabinoid system has been emerged as an endogenous therapeutic target to regulate inflammation after stroke insult, the downstream mechanisms and their potentials for therapeutic intervention remain controversial. Here we identified *trans*- and *cis*-hinokiresinols as novel non-selective antagonists for two G-protein-coupled cannabinoid receptors, cannabinoid receptor type 1 and type 2. The Electric Cell-substrate Impedance Sensing and Boyden chamber migration assays using primary microglial cultures revealed that both hinokiresinols significantly inhibited an endocannabinoid, 2-arachidonoylglycerol-induced migration. Hinokiresinols modulated 2-arachidonoylglycerol-induced mitochondrial bioenergetics in microglia as evidenced by inhibition of ATP turnover and reduction in respiratory capacity, thereby resulting in impaired migration activity. In rats subjected to transient middle cerebral artery occlusion (1.5-h) followed by 24-h reperfusion, post-ischemic treatment with hinokiresinols (2 and 7-h after the onset of ischemia, 10 mg/kg) significantly reduced cerebral infarct and infiltration of ED1-positive microglial/macrophage cells into cerebral ischemic lesions *in vivo*. Co-administration of exogenous 2-AG (1 mg/kg, i.v., single dose at 2 h after starting MCAO) abolished the protective effect of *trans*-hinokiresionol. These results suggest that hinokiresinols may serve as stroke treatment by targeting the endocannabinoid system. Alteration of mitochondrial bioenergetics and consequent inhibition of inflammatory cells migration may be a novel mechanism underlying anti-ischemic effects conferred by cannabinoid receptor antagonists.

## Introduction

Emerging evidence supports a key role of the endocannabinoid system in ischemic stroke, especially in modulation of post-ischemic inflammation [1–3]. The endocannabinoid system comprises cannabinoid receptors, their endogenous ligands [*e*.*g*., endocannabinoids such as anandamide and 2-arachidonoylglycerol (2-AG)], specific transporters, as well as their metabolizing enzymes. The cannabinoid receptors incorporate G protein-coupled type 1 cannabinoid receptors (CB1Rs), type 2 receptors (CB2Rs), as well as non-CB1/CB2Rs. The functional roles of cannabinoid receptors are often poorly estimated, mainly due to unsegregated expressions of CB1Rs, CB2Rs and some non-canonical cannabinoid receptors in various cell types, as well as improper dose and/or insufficient selectivity of ligands. Nonetheless, recent studies suggest a neuroprotective role of the cannabinoid signaling system in cerebral ischemia [1–3]. For example, during ischemic injury endocannabinoids accumulate, cannabinoid receptors are up-regulated, and treatment with cannabinoid receptor modulators protects neurons against ischemic damage [1–3]. Interestingly, the endocannabinoid system has been reported to exert modulatory actions on resident microglial cells and infiltrated inflammatory cells in the brain [4–7]. However, the underlying mechanism remains largely unknown.

Naturally occurring phenylpropanoids, *cis*- and *trans*-hinokiresinols, are major components in the essential oil extracted from various Liliaceae plants and have been reported to exhibit anti-oxidant, anti-inflammatory, anti-apoptotic and estrogen-like activities [8–11]. These pharmacological properties would be beneficial in treating cerebral ischemia. Indeed, we recently demonstrated that post-ischemic treatment of hinokiresinols significantly attenuated infarct volumes and neurological impairments in a rat model of transient cerebral ischemia [12]. Interestingly, hinokiresinols share structural characteristics with some phytocannabinoids such as *trans*-caryophyllene, falcarinol, rutamarin [13] and naturally prenylated resveratrol analogs, *trans*-arachidin-1 and -3 [14] identified beyond the cannabis plants. Based on the structural similarity of hinokiresinols with these plant-originated CB1R/CB2R regulators and their anti-inflammatory effects, we here investigated whether hinokiresinols selectively bind to cannabinoid receptors and act as selective agonists/antagonists. Using a rat model with transient middle cerebral artery occlusion (tMCAO), we further investigated whether hinokiresinols can afford neuroprotection by modulating microglia/macrophages accumulation in ischemic lesions.

## Materials and Methods

### Antibodies and reagents

Mouse anti-ED1 antibody was purchased from Serotec (Oxford, UK) and alexa 555-conjugated goat anti-mouse antibody from Molecular Probes (Eugene, OR). 2-AG was purchased from Cayman Chemical (Ann Arbor, MI). Oligomycin, carbonyl cyanide p-[trifluoromethoxy]-phenyl-hydrazone (FCCP), and rotenone, Hoechst 33258, and all other drugs were obtained from Sigma–Aldrich (St. Louis, MO). Stereo-specific syntheses of *trans*- and *cis*-hinokiresinols were performed as previously described with minor modifications [15]. The purity (98%) and absolute configuration were resolved using HPLC.

### Animals

Male Sprague-Dawley (SD) rats weighing between 260 and 300 g were purchased from Charles River Laboratories (Seoul, Korea) and maintained on a 12-h light/dark cycle with *ad libitum* access to food and water. Rats were acclimated to their environment for a minimum of 2 days prior to use. All experimental procedures involving animals were performed in accordance with the NIH Guide for the Care and Use of Laboratory Animals and were approved by Korea University Institutional Animal Care & Use Committee (KUIACUC-20121226-1, KUIACUC-20131218-4&6, KUIACUC-20130104-2). All efforts were made to minimize animal suffering, reduce the number of animals used, and utilize alternatives to in vivo techniques, if available.

### Competition Receptor Binding assay

Increasing concentrations of hinokiresinols were incubated with 0.2 nM of the non-selective CB1/CB2 agonist [^3^H]-CP-55,940 in a final volume of 1 mL of binding buffer (50 mM Tris, 0.05% bovine serum albumin, 5 mM of MgCl_2_, pH 7.4) as described previously [16]. Each binding assay contained 100 or 25 μg of membrane protein fractions prepared from mouse brain or CHO-human CB2R (hCB2R) cells, respectively. Non-specific binding was defined as binding observed in the presence of 10 μM of the non-selective CB1/CB2 ligand WIN-55,212–2. Reactions were carried out for 90 min at room temperature and terminated by rapid vacuum filtration through Whatman GF/B glass fiber filters followed by three washes with ice-cold binding buffer. Analysis of the binding data was performed using the non-linear regression (Curve Fit) function of GraphPad Prism® v4.0β to determine the IC_50_ value of the drug that displaced 50% of [^3^H]CP-55,940. A measure of affinity (K_i_) was derived from the IC_50_ values utilizing the Cheng-Prushoff equation [16]. As a positive control, competition binding was also conducted with a selective CB2 agonist AM-1241. As expected, AM-1241 produced full displacement of ^3^H-CP-55,940 from both receptors and exhibited more than 30-fold greater selectivity for hCB2R (Ki value of 6.6 nM) in relative to mCB1R (Ki value of 203 nM).

### Receptor Activity assay

For assessment of agonistic/antagonistic effects of hinokiresinols, serum response element (SRE)-luciferase reporter gene assay and forskolin-induced cAMP accumulation assays were performed using U2 OS cells stably expressing either CB1R or CB2R as previously described with some modifications [17].

### Cell migration assay

Pure microglial cells were prepared from primary mixed glial cell culture. Cerebral cortices from 1–2 days-old neonatal Sprague-Dawley rats were triturated to single cells. They were then plated into poly-D-lysine (1 μg/ml; Sigma-Aldrich, St. Louis, MO) coated 75 cm^2^ T-flask and maintained in MEM containing 10% fetal bovine serum. Seven or eight days after plating, microglia were detached from the flasks by mild shaking (37°C, 1 h at 200 rpm) and re-suspended in a desired cell density [18].

An automated Electric Cell-Substrate Impedance Sensing (ECIS)/taxis assay was performed as previously described with minor modification [19]. An ECIS/taxis array, which is composed of 8 chambers with lithographed paired surface electrodes within each chamber (8W Chemotaxis; Applied BioPhysics, Troy, NY), was pre-treated with a 10 mM cysteine solution for 15 min at room temperature. The autoclaved agarose solution (Seakem GTG; Cambrex, Rockland, ME) was mixed with pre-warmed (45–50°C) MEM to produce a 0.5% final concentration and loaded into the array chamber. After the agarose had solidified, wells for chemoattractant and cells were cut in the agarose layer using a pair of 14-gauge cannulas (about 1.8 mm O.D.), spaced 1 mm on either side of the target electrode. The agarose in the chamber arrays was then saturated with serum-free media for at least 5 minutes. Cells were added to the cell well (2.25×10^6^ cell/ml, 7 μl) and acclimated in a humidified CO_2_ incubator. After one hour, a chemoattractant 2-AG (100 nM) was loaded, connected to the ECIS instrument (ECIS Z; Applied BioPhysics, Troy, NY) and the resistance to 1 volt AC at 4000 Hz was measured at 60 second intervals.

For quantitative analysis, a chemoattractant 2-AG (100 nM) was placed at the lower well of a chemotaxis chamber (Neuroprobe, Cabin John, MD) and microglia (4×10^5^ cells/ml in serum-free MEM) in the upper well were allowed to migrate to the lower well for 2 h, through membrane pore (8 μm pore). Non-migrated cells in the upper well were removed and migrated cells on the lower side filter were nuclei-stained with Harris hematoxylin and then counted using an inverted microscopy system (Leica DMIL and DFC420C camera, Leica, Nussloch, Germany).

### Oxygen consumption rate (OCR) assay

Live mitochondrial respiration was analyzed using the XF24 Extracellular Flux Analyzer (SeahorseBioscience, MA) as previously described [20]. In this assay, OCR, the rate of oxygen consumed by the cells, is an indicator for mitochondrial respiration, whereas the extracellular acidification rate (ECAR) reflects oxygen-independent glycolysis in the cells. OCR was assessed after sequential addition of drugs, oligomycin (an ATP synthase inhibitor), FCCP (protonophore), and rotenone (complex I inhibitor to completely block proton pumping via the electron transfer chain) to living microglia according to the manufacturer’s protocol and normalized to the baseline. Bioenergetic parameters were calculated as: 1) ATP turnover is the difference between the starting basal OCR and oligomycin-repressed OCR, and 2) Maximal respiration capacity is the difference in OCR between FCCP and rotenone exposure.

### Focal transient cerebral ischemia model

Rats were initially anesthetized with 3% isoflurane in a 70% N_2_O and 30% O_2_ (v/v) mixture via facemask and maintained with 2% isoflurane. Focal cerebral ischemia was achieved by right-sided endovascular middle cerebral artery occlusion (MCAO) as previously described [12]. Briefly, monofilament nylon suture (Ethicon) was inserted into the lumen of right external carotid artery stump and gently advanced 17.5 mm into the internal carotid artery to occlude the ostium of the MCAO. After 90 min of ischemia, the suture was released and the animal was allowed to recover. *Trans*- or *cis*-hinokiresinol (10 mg/kg) was dissolved in 5% DMSO/10% cremophor/sterile saline solution freshly before injection, and was administered intraperitoneally twice, as a post-ischemic treatment (at 2 h and 7 h after starting MCAO) in the presence or absence of 2-AG (1 mg/kg, i.v.; once at 2 h after the onset of MCAO). Larger exposure with 2-AG alone may have a neuroprotective effects [21, 22]. Thus, the dose and administration route of 2-AG was carefully chosen based on our preliminary studies to antagonize the effect of hinokiresinols on ischemic injury without its own *in vivo* efficacy. For the measurement of infarct volumes, the brain slices were stained with 2% 2,3,5-triphenyltetrazoliumchloride (TTC) at 37°C for 30 min to visualize the ischemic infarct. Infarct volumes were compensated for brain edema as we previously described [12]. All in vivo experiments and the subsequent data analysis were performed in a double-blinded and randomized manner.

### Immunocytochemistry

Cells were fixed in 4% paraformaldehyde and blocked with TBS supplemented with 5% normal goat serum for 1 h at room temperature. Cells were then stained with mouse anti-ED-1 (1:200) at 4°C overnight. After washing, cells were incubated with secondary antibody (2 μg/ml) for an additional 1 h at room temperature and counterstained with Hoechst 33258 for 20 min. Fluorescence was quantified using a fluorescence microscope (DM IL HC Fluo, Leica) equipped with a digital camera.

### Statistical analysis

Data were expressed as mean ± standard error of the mean (SEM) and analyzed for statistical significance by an analysis of variance (ANOVA) followed by the post-hoc Bonferroni test for multiple comparisons or by using non-parametric Kruskal-Wallis test followed by Mann-Whitney U test depending on the Levene test results (SPSS; release20.0.0.1, IBM Corp.). A *P* value <0.05 after Bonferroni correction was considered significant. The sample size was determined using a power calculations using G*power software (ver.3.1.7, Franz Faul, Universitat Kiel, Germany) assuming a 2-sided α-level of 0.05, 80% power, and t-test or one-way ANOVA with the mean and variance predicted from our previous studies [12, 23].

## Results

### Hinokiresinols act as CB1/CB2 receptor antagonists/inverse agonists

**Fig 1:**
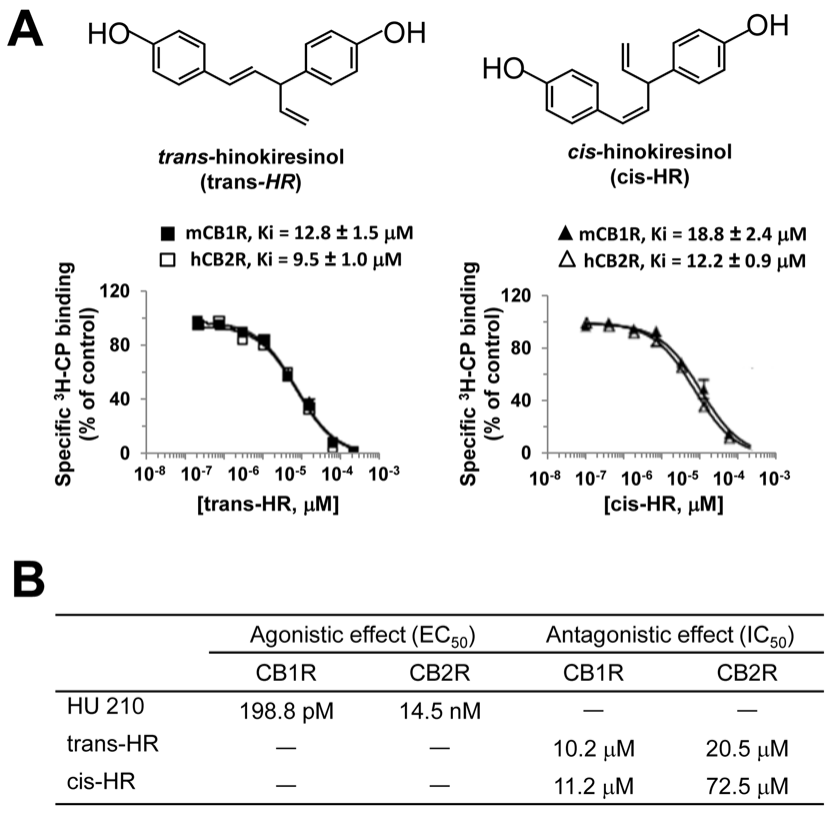
Selective binding of *trans*- and *cis*-hinokiresinols on CB1R and CB2Rs. (A) Competitive binding experiments against [^3^H]CP55,940 (^3^H-CP) were performed using either membrane fractions from mouse brain (mCB1R) or CHO cells stably expressing human CB2R (hCB2R) in the presence of *trans*- or *cis*-hinokiresinols (*trans*- or *cis*-HR). Data are presented as mean ± SEM. N = 6. (B) Calculated EC_50_ and IC_50_ values of antagonistic and agonistic activities from CB1R reporter gene assay and forskolin-stimulated cAMP accumulation assay in CHO-hCB2R cells. Hu-210 was used as a positive control for agonistic activity.

*Trans*- and *cis* -hinokiresinols specifically but non-selectively bind to both CB1Rs and CB2Rs with low molecular affinities and acted as weak receptor antagonists (Fig 1A and 1B). Thus, both *trans- and cis*-hinokiresinols bind to human CB2R (hCB2R) stably expressed in CHO cells with an apparent affinity (Ki) of 9.5 and 12.2 μM, respectively (Fig 1A). With similar affinities, *trans-* and *cis*-hinokiresinols also bind to murine CB1R (mCB1R) receptors with Ki values of 12.8 and 18.8 μM, respectively (Fig 1A). Interestingly, both hinokiresinols determined a complete displacement of ^3^H-CP-55,940 from both mCB1R and hCB2R (Fig 1A and 1B. Subsequent studies were conducted to determine the intrinsic activities of both hinokiresinols for each cannabinoid receptor subtypes. CB1R reporter gene assay and forskolin-stimulated cAMP accumulation assay in CHO-hCB2R cells demonstrated that administration with each hinokiresinol results in antagonism for CB1R and CB2R with IC_50_ values in micromolar ranges (Fig 1B). Pharmacokinetic data showed that low micromolar levels in blood could be achieved by 10 mg/kg injection in rats (data not shown).

### Hinokiresinols reduce microglial migration induced by 2-AG via modulating mitochondrial bioenergetics

**Fig 2:**
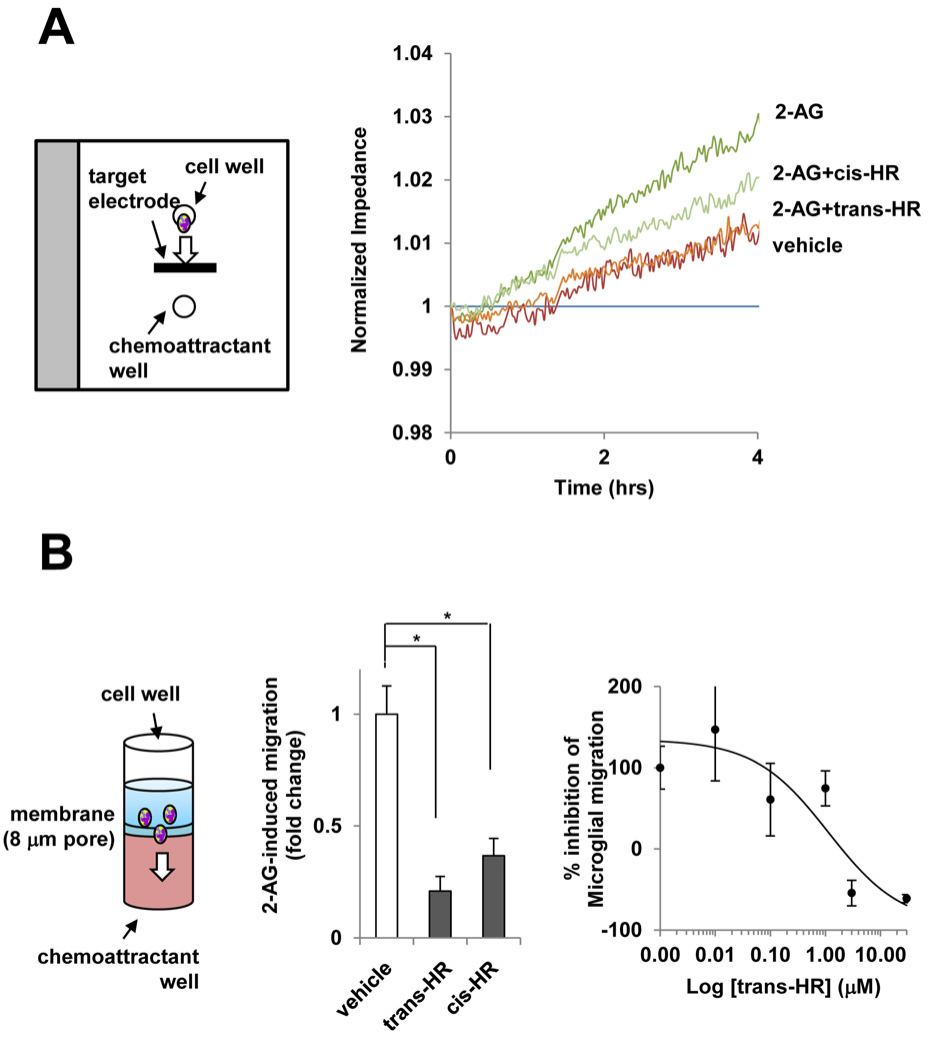
Hinokiresinols reduces 2-AG-induced microglial migration. (A) Schematic configuration of ECIS/taxis electrode design to measure an increase of resistance proportional to cell migration (left) and representative trace of ECIS change in the presence of hinokiresinols (right). The chemotactic response of pure primary microglia (1 X 10^4^ cells/well) was assessed using the linear target electrode, placed between cell and chemoattractant wells. (B) Principles of Boyden chemotaxis chamber (left). Microglial migration toward 2-AG-containing lower wells of the Boyden chamber in the absence or presence of hinokiresinols [10 μM each (middle) or indicated concentration of *trans*-hinokiresinols (right)] was quantified, and the results are expressed as fold change in numbers of migrated cells (left) or an inhibition percentage compared to basal migration and maximum migration by 2-AG (right), respectively. Values are mean ± SEM of 4–8 independent quantification of migration. ^*^ *P* < 0.05: vs. indicated group.

An endocannabinoid, 2-AG is an agonist without any marked CB1R/CB2R selectivity [2] and was shown to trigger microglial cell migration [7]. The Electric Cell-substrate Impedance Sensing (ECIS) and Boyden chamber migration assays revealed that both hinokiresinols inhibited 2-AG (100 nM)-induced microglial migration (Fig 2; 79.1% and 63.2% inhibition for *trans-* and *cis*-hinokiresinols, respectively; IC_50_ for *trans*-hinokiresinol = 0.502 μM).

**Fig 3:**
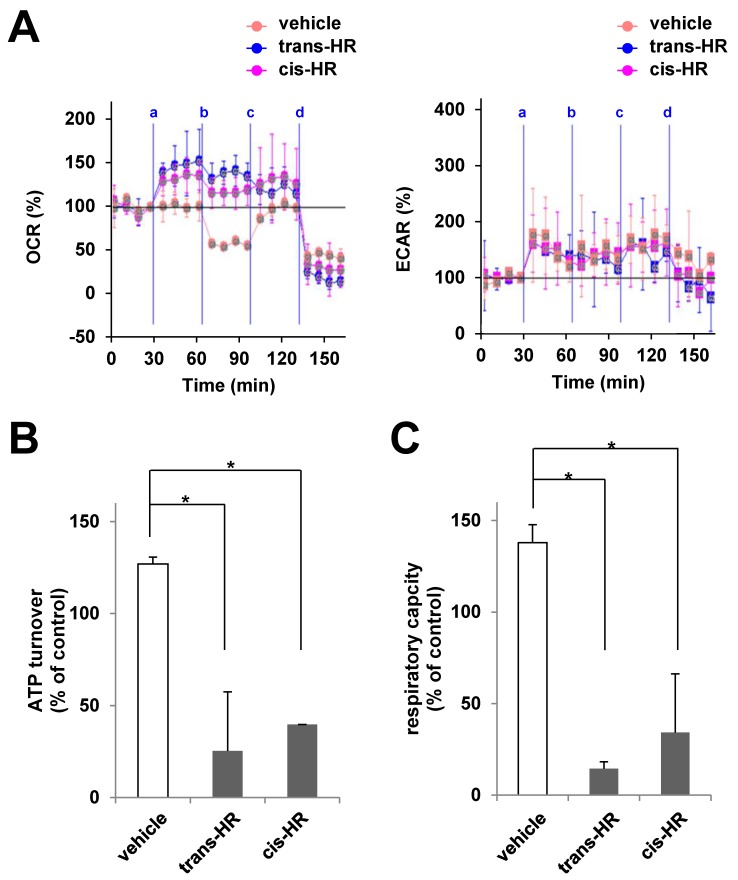
Hinokiresinols modulates 2-AG-induced mitochondrial bioenergetics in microglia. Pure microglia (5 X 10^5^ cells/ml, 150 μl/well) were treated with vehicle or hinokiresinols (HRs; line a), oligomycin (line b), FCCP (line c), and rotenone (line d) at indicated times and the rate of oxygen consumption (OCR, left) and extracellular acidification (ECAR, right) were recorded. (A) Real-time mitochondrial respiration was measured in microglia using the Seahorse analyzer. (B, C) ATP turnover and respiration capacity were calculated based on OCR (A, left panel) and expressed as % of control. The data were normalized in vehicle controls and expressed as mean ± SEM (N = 4–8). ^*^ *P* <0.05: vs. indicated group.

Microglial motility was closely associated with the overall number, distribution, and bioenergetics status of mitochondria [24]. To assess the effects of hinokiresinols on 2-AG-induced mitochondrial bioenergetic functions, we determined real-time oxygen consumption rates (OCR, an indicator of oxidative phosphorylation) in the presence of oligomycin (an inhibitor of ATP synthase) to measure full ATP turnover, and then FCCP (an uncoupler of electron transport and oxidative phosphorylation) to measure the maximal respiratory capacity. We found that both hinokiresinols modulate 2-AG-induced mitochondrial respiratory bioenergetics (Fig 3), by reducing ATP turnover (80.0% and 68.7% inhibition for *trans*- and *cis*-hinokiresinols, respectively) and by exhausting respiratory capacity (89.5% and 75.2% inhibition for *trans*- and *cis* -hinokiresinols, respectively). Hinokiresinols alone did not significantly change the ATP turnover and respiratory capacity (data not shown). Neither of hinokiresinols significantly changed the ECAR, an indicator of glycolysis, regardless of the presence of 2-AG (Fig 3).

### Hinokiresinols reduce infiltration of ED1-positive microglial/macrophage cells to ischemic lesions

**Fig 4:**
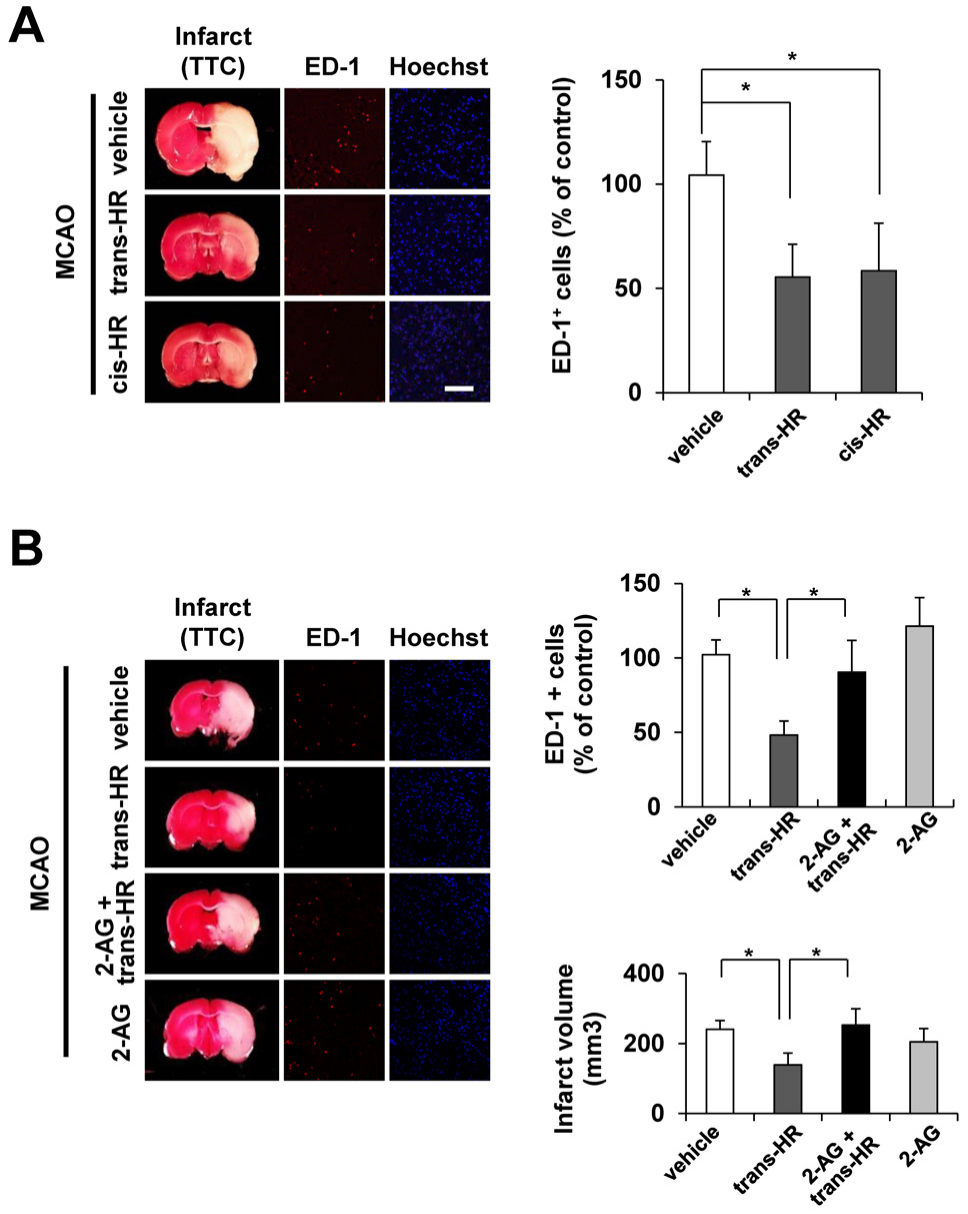
Post-treatment of hinokiresinols significantly reduced ED-1-postive microglia/macrophages in ischemic lesions. Rats were subject to MCAO for 90 min and then reperfused. After 24 h, coronal brain sections were stained with triphenyltetrazolium chloride (TTC) for brain infarct, anti-ED-1 antibody to detect macrophages/monocytes/activated microglia (red), or Hoechst for nuclei staining (blue). Data are presented as mean ± SEM. Scale bar = 50 μm. ^*^ *P* < 0.05: vs. indicated group. (A) *Trans-* or *cis*-hinokiresinol (10 mg/kg, i.p.) was administrated at 2 h and 7 h after the onset of MCAO. Representative images (left panel) and quantification of cell counts of ED-1-positive cells (right panel) are provided. N = 3–5. (B) Co-administration of 2-AG abolished the protective effect of *trans*-hinokiresionol. *Trans-* hinokiresinol was administrated in the presence or absence of 10 mg/kg 2-AG (once at 2 h after starting MCAO, i.v.). Representative images (left panel) and quantification (right) of either cell counts of ED-1-positive cells (upper) or infarct volume (lower) are provided. N = 4–7.

Previously, we found that hinokiresinols attenuated infarct size and edema in rats subjected to 1.5-h of MCAO, followed by 24-h of reperfusion [12]. In the present study, post-ischemic treatment with *trans*- and *cis*-hinokiresinols significantly reduced cerebral infarct volume, as assessed by TTC staining (Fig 4A; vehicle, 24.37 ± 0.91%; *trans*-hinokiresinol, 13.65 ± 1.55%; *cis*-hinokiresinol, 12.93 ± 1.73%). The treatment further reduced infiltration of ED1-positive microglial/macrophage cells into ischemic lesions (Fig 4A; 46.8% and 43.9% inhibition for *trans-* and *cis*-hinokiresinols, respectively). Co-administration with 2-AG significantly abolished inhibitory effects of *trans*-hinokiresinol on microglial/macrophageal migration (Fig 4B; ED-1-positive cell counts for vehicle-treated group, 102.17 ± 9.90%; *trans*-hinokiresinol only, 48.25 ± 9.32%; both 2-AG and *trans*-hinokiresinol, 90.35 ± 21.34%; 2-AG only, 121.36 ± 19.15%). Co-administration of 2-AG also reduced the protective effect of *trans*-hinokiresinol (Fig 4B), as shown in changes in infarct volume (lower right panel; vehicle-treated group, 240.29 ± 12.53 mm^3^; *trans*-hinokiresinol only, 138.96 ± 19.25 mm^3^; both 2-AG and *trans*-hinokiresinol, 252.71 ±23.39 mm^3^; 2-AG only, 204.95 ± 18.86 mm^3^) and edema volume (data not shown; vehicle, 12.74 ± 1.22%; *trans*-hinokiresinol only, 4.83 ± 1.44%; both 2-AG and *trans*-hinokiresinol, 10.76 ± 2.33%; 2-AG only, 7.18 ± 1.26%). 2-AG itself did not show significant neuroprotective effect on infarct and edema volume in MCAO rats at the dosing regimen chosen in the current study (1 mg/kg, i.v. once at 2 h after starting; Fig 4B). Sham operation or treatment with hinokiresinol in the absence of transient ischemia did not induce any microglial/macrophage infiltration (data not shown).

## Discussion

Inflammatory responses constitute key events in the pathophysiology of cerebral ischemic stroke, including increased release of cytotoxic cytokines, activation of residual astrocytes and microglia, and adhesion and infiltration of peripheral inflammatory cells due to BBB damage. Previously, we and other researchers demonstrated that inhibition of infiltration of peripheral inflammatory cells into ischemic lesions reduced ischemic brain damage in animal models of ischemic stroke [6, 18, 25].

The endocannabinoid system is considered as a major modulator of not only neuronal survival, but also neuro-inflammation. Much evidence obtained from various *in vivo* and *in vitro* studies suggests a neuroprotective role of the cannabinoid receptors in ischemic brain [1]. At this regard, the endocannabinoid system draws much attention as highly promising therapeutic compounds for the treatment of cerebral ischemic injury than ever. For example, an endocannabinoid 2-AG is generated by neurons, astrocytes, and microglial cells, and dramatically increased in pathological stimuli such as neuroinflammation in ischemic stroke [26]. Although it is previously believed that peripheral immune cells predominantly express CB2Rs, recent data also support the increased expression and functional role of peripheral CB1Rs in ischemic stroke [27]. In activated microglia/macrophages, both CB1R and CB2R are expressed but at different subcellular locations [7]. Dynamic changes in the subcellular location of cannabinoid receptors might be associated with functional changes. For example, CB2Rs are accumulated at the leading edges of activated microglial cells upon migration stimuli [7]. However, the downstream mechanisms of CBRs in the microglial/macrophage motility never been thoroughly elucidated.

The present study supports a crucial role of mitochondrial bioenergetics in endocannabinoid-induced microglial migration. We found for the first time that *trans-* and *cis-*hinokiresinols specifically bind to cannabinoid receptors and act as antagonists for both CB1R and CB2R (Fig 1). Consequently, both hinokiresinols suppress the 2-AG-induced migration of microglial cells. Mitochondrial respiration is required for migration of immunocytes, and mitochondria redistribute towards the uropod of polarized migrating immune cells by a process involving shape rearrangements (*e*.*g*., fission, fusion) [24]. Our data show that hinokiresinols inhibit 2-AG-induced mitochondrial ATP turnover and exhaust respiratory capacity in microglia (Fig 3). Therefore, hinokiresinols may inhibit 2-AG-induced microglial migration via suppression of mitochondrial bioenergetics. In addition, both hinokiresinol isomers inhibit the *in vivo* recruitment of ED-1 positive microglia/macrophages to ischemic lesions in MCAO rats (Fig 4A). The inhibition of microglial/macrophageal migration and associated ischemic brain injury are significantly abolished by exogenous 2-AG (Fig 4B). Our data supports the notion that the endocannabinoid system plays a key role in ischemic stroke and that the inhibition of endocannabinoid signaling in the peripheral inflammatory cells might provide neuroprotection in ischemic stroke. Non-selective CBR antagonists, hinokiresinols modulate mitochondrial bioenergetics and migration ability of microglia, resulting in significant inhibition of inflammatory cells infiltration into ischemic lesions, and thus leading to neuroprotection. Although the downstream signaling of hinokiresinols remains to be addressed with detailed studies, our studies suggested that hinokiresinols are a promising candidate for stroke treatment without producing psychoactive side effects associated with CB1R activation.

It has been shown that endo-/phyto-cannabinoids affects the mitochondrial activity in brain, white adipose tissue, muscle, and liver [28, 29]. However, whether and how the endocannabinoid system affect microglial/macrophageal mitochondria are poorly understood. Mitochondrial effects of endocannabinoids have been interpreted either as CB1R or non-receptor-mediated alteration of mitochondrial membranes. The highly lipophilic nature of endocannabinoids also allows them to directly access mitochondria. A recent report showed that CB1Rs are present in mitochondrial membranes in neurons, where their activation results in decreased cAMP concentration and complex I activity [30]. Although our data suggest the effects of 2-AG on mitochondrial bioenergetics in microglia/macrophage, especially in migration activity, studies that address the detailed molecular basis of this response and the engagement of cannabinoid receptors in this context remain to be performed.

In summary, our data showed that *trans*- and *cis*-hinokiresinols provide neuroprotective effects during cerebral ischemic insult. Hinokiresinols significantly inhibit the infiltration of peripheral microglia/macrophages into cerebral ischemic lesion, potentially via modulating their mitochondrial bioenergetics and migratory activities. The present study adds support to a key role of the endocannabinoid system in the post-ischemic inflammation, and provides new insights for the development of drugs to limit infiltration of inflammatory cells into ischemic lesions after stroke.

## Supporting Information

S1 ARRIVE Checklist (PDF)

## Abbreviations

2-AG: 2-arachidonoylglycerol
CB1R: cannabinoid receptor type 1
CB2R: cannabinoid receptor type 2
ECAR: extracellular acidification rate
ECIS: Electric Cell-substrate Impedance Sensing
FCCP: carbonyl cyanide p-[trifluoromethoxy]-phenyl-hydrazone
MCAO: middle cerebral artery occlusion
OCR: Oxygen consumption rate
TTC: 2,3,5-triphenyltetrazoliumchloride
